# Supplementary material for: Modeling glioblastoma heterogeneity as a dynamic network of cell states
Source: Mol Syst Biol. 2021 Sep 16;17(9):e10105. doi: 10.15252/msb.202010105 (PMC8444284; doi:10.15252/msb.202010105)
Supplement: Supplementary file 5 — Source Data for Figure 3 [file MSB-17-e10105-s001.zip › Figure3A_sourcedata/GSEA_3065/nosigdb_state6.GseaPreranked.1622718443993/VANGRONINGEN_MES.html]

Details for gene set VANGRONINGEN\_MES[GSEA]

|  || Dataset | state6 |
| Phenotype | NoPhenotypeAvailable |
| Upregulated in class | na\_neg |
| GeneSet | VANGRONINGEN\_MES |
| Enrichment Score (ES) | -0.5407126 |
| Normalized Enrichment Score (NES) | -2.5486345 |
| Nominal p-value | 0.0 |
| FDR q-value | 0.0 |
| FWER p-Value | 0.0 |
Table: GSEA Results Summary

  

Fig 1: Enrichment plot: VANGRONINGEN\_MES      
 Profile of the Running ES Score & Positions of GeneSet Members on the Rank Ordered List

  

| PROBE | GENE SYMBOL | GENE\_TITLE | RANK IN GENE LIST | RANK METRIC SCORE | RUNNING ES | CORE ENRICHMENT || 1 | RAB31 |  |  | 23 | 0.284 | 0.0051 | No |
| 2 | PHLDA3 |  |  | 29 | 0.272 | 0.0119 | No |
| 3 | PLK2 |  |  | 43 | 0.249 | 0.0172 | No |
| 4 | PEA15 |  |  | 79 | 0.225 | 0.0193 | No |
| 5 | SOX9 |  |  | 148 | 0.193 | 0.0165 | No |
| 6 | FILIP1L |  |  | 175 | 0.183 | 0.0184 | No |
| 7 | KLF10 |  |  | 234 | 0.166 | 0.0161 | No |
| 8 | WWTR1 |  |  | 241 | 0.164 | 0.0199 | No |
| 9 | KDM5B |  |  | 254 | 0.161 | 0.0229 | No |
| 10 | ZFP36L1 |  |  | 266 | 0.159 | 0.0259 | No |
| 11 | NR3C1 |  |  | 377 | 0.139 | 0.0167 | No |
| 12 | RAP1A |  |  | 411 | 0.134 | 0.0165 | No |
| 13 | SVIL |  |  | 471 | 0.126 | 0.0130 | No |
| 14 | DESI2 |  |  | 511 | 0.122 | 0.0117 | No |
| 15 | DUSP14 |  |  | 592 | 0.115 | 0.0054 | No |
| 16 | TMEM263 |  |  | 690 | 0.107 | -0.0032 | No |
| 17 | LITAF |  |  | 692 | 0.106 | -0.0004 | No |
| 18 | SH3BGRL |  |  | 701 | 0.105 | 0.0015 | No |
| 19 | SQSTM1 |  |  | 719 | 0.104 | 0.0024 | No |
| 20 | PALLD |  |  | 752 | 0.102 | 0.0014 | No |
| 21 | RHOC |  |  | 794 | 0.099 | -0.0008 | No |
| 22 | ZNF217 |  |  | 798 | 0.098 | 0.0016 | No |
| 23 | MEOX2 |  |  | 803 | 0.098 | 0.0038 | No |
| 24 | QKI |  |  | 820 | 0.097 | 0.0045 | No |
| 25 | ERBIN |  |  | 831 | 0.096 | 0.0060 | No |
| 26 | RAB13 |  |  | 858 | 0.094 | 0.0055 | No |
| 27 | GDF15 |  |  | 888 | 0.092 | 0.0046 | No |
| 28 | ARHGAP1 |  |  | 897 | 0.091 | 0.0061 | No |
| 29 | LIX1L |  |  | 908 | 0.090 | 0.0074 | No |
| 30 | GNS |  |  | 978 | 0.087 | 0.0016 | No |
| 31 | NBR1 |  |  | 1004 | 0.086 | 0.0010 | No |
| 32 | RAB29 |  |  | 1012 | 0.085 | 0.0025 | No |
| 33 | MBD2 |  |  | 1041 | 0.083 | 0.0015 | No |
| 34 | RGS3 |  |  | 1074 | 0.082 | -0.0001 | No |
| 35 | FAM120A |  |  | 1110 | 0.080 | -0.0020 | No |
| 36 | CTDSP2 |  |  | 1111 | 0.080 | 0.0002 | No |
| 37 | HIBADH |  |  | 1125 | 0.079 | 0.0008 | No |
| 38 | PEAK1 |  |  | 1141 | 0.078 | 0.0012 | No |
| 39 | FZD2 |  |  | 1178 | 0.076 | -0.0010 | No |
| 40 | ERRFI1 |  |  | 1210 | 0.075 | -0.0026 | No |
| 41 | SYDE1 |  |  | 1260 | 0.073 | -0.0064 | No |
| 42 | ID3 |  |  | 1329 | 0.070 | -0.0125 | No |
| 43 | CAPN2 |  |  | 1385 | 0.068 | -0.0172 | No |
| 44 | JAK1 |  |  | 1393 | 0.068 | -0.0162 | No |
| 45 | IFITM3 |  |  | 1398 | 0.068 | -0.0148 | No |
| 46 | KIF13A |  |  | 1399 | 0.067 | -0.0129 | No |
| 47 | KLF6 |  |  | 1424 | 0.067 | -0.0140 | No |
| 48 | RBMS1 |  |  | 1513 | 0.063 | -0.0227 | No |
| 49 | SHROOM3 |  |  | 1587 | 0.061 | -0.0296 | No |
| 50 | TFE3 |  |  | 1605 | 0.060 | -0.0300 | No |
| 51 | TJP1 |  |  | 1659 | 0.058 | -0.0347 | No |
| 52 | MBNL1 |  |  | 1705 | 0.057 | -0.0385 | No |
| 53 | OLFML2A |  |  | 1756 | 0.055 | -0.0429 | No |
| 54 | MEST |  |  | 1882 | 0.051 | -0.0563 | No |
| 55 | STK38L |  |  | 1886 | 0.051 | -0.0553 | No |
| 56 | SKIL |  |  | 1921 | 0.050 | -0.0579 | No |
| 57 | ACADVL |  |  | 1940 | 0.049 | -0.0587 | No |
| 58 | TRIM5 |  |  | 1964 | 0.049 | -0.0601 | No |
| 59 | ANXA5 |  |  | 1978 | 0.048 | -0.0603 | No |
| 60 | C1orf198 |  |  | 1998 | 0.048 | -0.0613 | No |
| 61 | CLIC4 |  |  | 2035 | 0.047 | -0.0643 | No |
| 62 | SUCLG2 |  |  | 2110 | 0.044 | -0.0718 | No |
| 63 | TCF7L2 |  |  | 2133 | 0.044 | -0.0732 | No |
| 64 | LASP1 |  |  | 2161 | 0.043 | -0.0752 | No |
| 65 | SPRY1 |  |  | 2167 | 0.043 | -0.0747 | No |
| 66 | RGS10 |  |  | 2208 | 0.041 | -0.0783 | No |
| 67 | ZCCHC24 |  |  | 2231 | 0.040 | -0.0798 | No |
| 68 | UAP1 |  |  | 2254 | 0.040 | -0.0813 | No |
| 69 | UGDH |  |  | 2261 | 0.040 | -0.0809 | No |
| 70 | ACAP2 |  |  | 2265 | 0.040 | -0.0802 | No |
| 71 | GNG12 |  |  | 2317 | 0.038 | -0.0852 | No |
| 72 | RAP1B |  |  | 2322 | 0.038 | -0.0846 | No |
| 73 | SSBP4 |  |  | 2364 | 0.037 | -0.0885 | No |
| 74 | ID1 |  |  | 2451 | 0.035 | -0.0977 | No |
| 75 | RIT1 |  |  | 2457 | 0.035 | -0.0974 | No |
| 76 | CMTM3 |  |  | 2459 | 0.035 | -0.0965 | No |
| 77 | PYGL |  |  | 2462 | 0.035 | -0.0958 | No |
| 78 | STAT1 |  |  | 2471 | 0.034 | -0.0958 | No |
| 79 | GPR137B |  |  | 2538 | 0.032 | -0.1028 | No |
| 80 | POLR2L |  |  | 2539 | 0.032 | -0.1019 | No |
| 81 | ITPRIPL2 |  |  | 2585 | 0.031 | -0.1064 | No |
| 82 | ELF1 |  |  | 2696 | 0.029 | -0.1186 | No |
| 83 | VIM |  |  | 2831 | 0.026 | -0.1338 | No |
| 84 | FN1 |  |  | 2839 | 0.026 | -0.1339 | No |
| 85 | SDF4 |  |  | 2876 | 0.025 | -0.1375 | No |
| 86 | MYADM |  |  | 2933 | 0.023 | -0.1435 | No |
| 87 | MAML2 |  |  | 2940 | 0.023 | -0.1436 | No |
| 88 | HOMER1 |  |  | 2950 | 0.023 | -0.1440 | No |
| 89 | SDCBP |  |  | 2964 | 0.023 | -0.1449 | No |
| 90 | MGAT2 |  |  | 3009 | 0.022 | -0.1496 | No |
| 91 | ARPC1B |  |  | 3027 | 0.021 | -0.1510 | No |
| 92 | TNFRSF1A |  |  | 3049 | 0.021 | -0.1529 | No |
| 93 | AMMECR1 |  |  | 3112 | 0.020 | -0.1597 | No |
| 94 | ARL4A |  |  | 3127 | 0.019 | -0.1608 | No |
| 95 | SDC4 |  |  | 3129 | 0.019 | -0.1604 | No |
| 96 | REST |  |  | 3154 | 0.019 | -0.1628 | No |
| 97 | CETN2 |  |  | 3175 | 0.018 | -0.1646 | No |
| 98 | MAN2A1 |  |  | 3233 | 0.017 | -0.1709 | No |
| 99 | SNAP23 |  |  | 3236 | 0.017 | -0.1707 | No |
| 100 | GNAI1 |  |  | 3240 | 0.017 | -0.1706 | No |
| 101 | YAP1 |  |  | 3295 | 0.016 | -0.1766 | No |
| 102 | MOB1A |  |  | 3364 | 0.014 | -0.1843 | No |
| 103 | ACTN1 |  |  | 3411 | 0.013 | -0.1894 | No |
| 104 | CNN3 |  |  | 3415 | 0.013 | -0.1894 | No |
| 105 | MBTPS1 |  |  | 3531 | 0.011 | -0.2027 | No |
| 106 | TGFB1I1 |  |  | 3568 | 0.010 | -0.2067 | No |
| 107 | HSPB1 |  |  | 3587 | 0.010 | -0.2086 | No |
| 108 | SHC1 |  |  | 3621 | 0.009 | -0.2123 | No |
| 109 | TPM1 |  |  | 3680 | 0.007 | -0.2190 | No |
| 110 | ARMCX2 |  |  | 3696 | 0.007 | -0.2206 | No |
| 111 | PXDC1 |  |  | 3715 | 0.006 | -0.2225 | No |
| 112 | QSOX1 |  |  | 3763 | 0.005 | -0.2279 | No |
| 113 | CRELD2 |  |  | 3839 | 0.004 | -0.2367 | No |
| 114 | MYL12B |  |  | 3900 | 0.002 | -0.2438 | No |
| 115 | LPP |  |  | 3941 | 0.002 | -0.2485 | No |
| 116 | RGL1 |  |  | 3970 | 0.001 | -0.2518 | No |
| 117 | ATP10D |  |  | 3992 | 0.001 | -0.2542 | No |
| 118 | TPM2 |  |  | 4007 | 0.001 | -0.2559 | No |
| 119 | ANTXR1 |  |  | 4077 | -0.001 | -0.2640 | No |
| 120 | SPRY4 |  |  | 4103 | -0.001 | -0.2670 | No |
| 121 | EHD2 |  |  | 4108 | -0.001 | -0.2674 | No |
| 122 | CBFB |  |  | 4209 | -0.003 | -0.2792 | No |
| 123 | AEBP1 |  |  | 4222 | -0.004 | -0.2805 | No |
| 124 | WIPI1 |  |  | 4237 | -0.004 | -0.2821 | No |
| 125 | SMAD3 |  |  | 4246 | -0.004 | -0.2829 | No |
| 126 | SERPINE2 |  |  | 4250 | -0.004 | -0.2831 | No |
| 127 | DCAF6 |  |  | 4252 | -0.004 | -0.2831 | No |
| 128 | TSC22D2 |  |  | 4274 | -0.005 | -0.2855 | No |
| 129 | PDGFC |  |  | 4280 | -0.005 | -0.2860 | No |
| 130 | FAM129A |  |  | 4300 | -0.005 | -0.2881 | No |
| 131 | TUBB6 |  |  | 4313 | -0.005 | -0.2894 | No |
| 132 | NANS |  |  | 4323 | -0.005 | -0.2903 | No |
| 133 | SPRED1 |  |  | 4403 | -0.007 | -0.2995 | No |
| 134 | RHOJ |  |  | 4595 | -0.011 | -0.3218 | No |
| 135 | ELK4 |  |  | 4672 | -0.012 | -0.3305 | No |
| 136 | ABRACL |  |  | 4687 | -0.012 | -0.3319 | No |
| 137 | ITGAV |  |  | 4722 | -0.013 | -0.3355 | No |
| 138 | L3HYPDH |  |  | 4728 | -0.013 | -0.3358 | No |
| 139 | FIBIN |  |  | 4733 | -0.013 | -0.3359 | No |
| 140 | SASH1 |  |  | 4774 | -0.014 | -0.3402 | No |
| 141 | SEC14L1 |  |  | 4793 | -0.014 | -0.3420 | No |
| 142 | C6orf120 |  |  | 4817 | -0.015 | -0.3443 | No |
| 143 | PTPN14 |  |  | 4849 | -0.015 | -0.3476 | No |
| 144 | NOTCH2 |  |  | 4874 | -0.016 | -0.3500 | No |
| 145 | TMBIM4 |  |  | 4900 | -0.017 | -0.3525 | No |
| 146 | RNH1 |  |  | 4982 | -0.018 | -0.3616 | No |
| 147 | GRN |  |  | 4995 | -0.018 | -0.3625 | No |
| 148 | STAT3 |  |  | 5004 | -0.018 | -0.3630 | No |
| 149 | LRP10 |  |  | 5011 | -0.018 | -0.3632 | No |
| 150 | ADGRG6 |  |  | 5012 | -0.019 | -0.3627 | No |
| 151 | IQGAP2 |  |  | 5055 | -0.019 | -0.3671 | No |
| 152 | MYL12A |  |  | 5109 | -0.021 | -0.3728 | No |
| 153 | SFT2D2 |  |  | 5173 | -0.022 | -0.3797 | No |
| 154 | LIPA |  |  | 5191 | -0.022 | -0.3811 | No |
| 155 | LAMP1 |  |  | 5265 | -0.024 | -0.3891 | No |
| 156 | NFIC |  |  | 5317 | -0.025 | -0.3945 | No |
| 157 | PDLIM1 |  |  | 5379 | -0.026 | -0.4010 | No |
| 158 | PLS3 |  |  | 5389 | -0.026 | -0.4014 | No |
| 159 | DDR2 |  |  | 5406 | -0.026 | -0.4026 | No |
| 160 | OGFRL1 |  |  | 5408 | -0.027 | -0.4019 | No |
| 161 | SCRG1 |  |  | 5450 | -0.027 | -0.4061 | No |
| 162 | PPIC |  |  | 5491 | -0.028 | -0.4100 | No |
| 163 | DPY19L1 |  |  | 5623 | -0.031 | -0.4247 | No |
| 164 | SFT2D1 |  |  | 5625 | -0.031 | -0.4240 | No |
| 165 | SPATA20 |  |  | 5627 | -0.031 | -0.4232 | No |
| 166 | THBS1 |  |  | 5670 | -0.032 | -0.4273 | No |
| 167 | PTPRK |  |  | 5681 | -0.032 | -0.4276 | No |
| 168 | IFITM2 |  |  | 5741 | -0.033 | -0.4337 | No |
| 169 | ITM2C |  |  | 5766 | -0.034 | -0.4356 | No |
| 170 | ANXA6 |  |  | 5817 | -0.035 | -0.4406 | No |
| 171 | CTNNA1 |  |  | 5850 | -0.035 | -0.4434 | No |
| 172 | F2R |  |  | 5881 | -0.036 | -0.4460 | No |
| 173 | CMTM6 |  |  | 5884 | -0.036 | -0.4453 | No |
| 174 | GALNT10 |  |  | 5939 | -0.037 | -0.4507 | No |
| 175 | CSRP1 |  |  | 5955 | -0.037 | -0.4514 | No |
| 176 | HIPK3 |  |  | 5971 | -0.037 | -0.4522 | No |
| 177 | HS3ST3A1 |  |  | 5988 | -0.038 | -0.4531 | No |
| 178 | CREB3L2 |  |  | 5989 | -0.038 | -0.4520 | No |
| 179 | MRC2 |  |  | 6024 | -0.039 | -0.4550 | No |
| 180 | HYOU1 |  |  | 6028 | -0.039 | -0.4543 | No |
| 181 | PLSCR1 |  |  | 6134 | -0.041 | -0.4656 | No |
| 182 | PLEKHA2 |  |  | 6149 | -0.041 | -0.4661 | No |
| 183 | CXCL12 |  |  | 6173 | -0.042 | -0.4677 | No |
| 184 | PHLDB2 |  |  | 6234 | -0.043 | -0.4736 | No |
| 185 | SEPT10 |  |  | 6275 | -0.044 | -0.4772 | No |
| 186 | COPA |  |  | 6322 | -0.045 | -0.4814 | No |
| 187 | GAS1 |  |  | 6366 | -0.046 | -0.4852 | No |
| 188 | EPS8 |  |  | 6447 | -0.048 | -0.4934 | No |
| 189 | KDELC2 |  |  | 6471 | -0.049 | -0.4948 | No |
| 190 | EGR1 |  |  | 6519 | -0.050 | -0.4990 | No |
| 191 | CTSO |  |  | 6552 | -0.052 | -0.5013 | No |
| 192 | DSE |  |  | 6569 | -0.052 | -0.5018 | No |
| 193 | SDC2 |  |  | 6588 | -0.053 | -0.5025 | No |
| 194 | FZD7 |  |  | 6710 | -0.056 | -0.5153 | No |
| 195 | TGFBR2 |  |  | 6727 | -0.057 | -0.5156 | No |
| 196 | IL13RA1 |  |  | 6748 | -0.057 | -0.5164 | No |
| 197 | DNAJC10 |  |  | 6757 | -0.058 | -0.5158 | No |
| 198 | FNDC3B |  |  | 6767 | -0.058 | -0.5153 | No |
| 199 | RNFT1 |  |  | 6852 | -0.060 | -0.5236 | No |
| 200 | GJA1 |  |  | 6876 | -0.061 | -0.5246 | No |
| 201 | SLC16A4 |  |  | 6902 | -0.062 | -0.5259 | No |
| 202 | SLC38A2 |  |  | 6934 | -0.063 | -0.5279 | No |
| 203 | ADGRE5 |  |  | 6952 | -0.063 | -0.5281 | No |
| 204 | IGFBP5 |  |  | 7011 | -0.065 | -0.5332 | No |
| 205 | MMP2 |  |  | 7016 | -0.065 | -0.5319 | No |
| 206 | TSPAN4 |  |  | 7026 | -0.066 | -0.5312 | No |
| 207 | PPT1 |  |  | 7033 | -0.066 | -0.5301 | No |
| 208 | IFI16 |  |  | 7041 | -0.066 | -0.5291 | No |
| 209 | ELK3 |  |  | 7080 | -0.067 | -0.5318 | No |
| 210 | LIFR |  |  | 7101 | -0.068 | -0.5323 | No |
| 211 | FAM3C |  |  | 7110 | -0.068 | -0.5313 | No |
| 212 | DNAJC1 |  |  | 7129 | -0.069 | -0.5316 | No |
| 213 | SCPEP1 |  |  | 7146 | -0.069 | -0.5316 | No |
| 214 | LRRC17 |  |  | 7166 | -0.070 | -0.5319 | No |
| 215 | LEPROT |  |  | 7199 | -0.071 | -0.5338 | No |
| 216 | DUSP6 |  |  | 7215 | -0.071 | -0.5336 | No |
| 217 | WNT5A |  |  | 7276 | -0.074 | -0.5387 | Yes |
| 218 | ARL1 |  |  | 7285 | -0.074 | -0.5376 | Yes |
| 219 | TOR1AIP1 |  |  | 7287 | -0.074 | -0.5357 | Yes |
| 220 | PTBP1 |  |  | 7293 | -0.074 | -0.5343 | Yes |
| 221 | HES1 |  |  | 7301 | -0.074 | -0.5330 | Yes |
| 222 | TFPI |  |  | 7359 | -0.076 | -0.5377 | Yes |
| 223 | LUZP1 |  |  | 7360 | -0.076 | -0.5356 | Yes |
| 224 | PLPP1 |  |  | 7391 | -0.078 | -0.5370 | Yes |
| 225 | SEL1L3 |  |  | 7402 | -0.078 | -0.5361 | Yes |
| 226 | NPC2 |  |  | 7426 | -0.079 | -0.5367 | Yes |
| 227 | ARHGEF40 |  |  | 7431 | -0.079 | -0.5350 | Yes |
| 228 | ERLIN1 |  |  | 7447 | -0.079 | -0.5346 | Yes |
| 229 | ENAH |  |  | 7451 | -0.079 | -0.5327 | Yes |
| 230 | CCDC80 |  |  | 7480 | -0.080 | -0.5339 | Yes |
| 231 | BAG3 |  |  | 7481 | -0.080 | -0.5316 | Yes |
| 232 | PRRX1 |  |  | 7482 | -0.081 | -0.5294 | Yes |
| 233 | SLC30A7 |  |  | 7487 | -0.081 | -0.5277 | Yes |
| 234 | PRCP |  |  | 7495 | -0.081 | -0.5263 | Yes |
| 235 | WLS |  |  | 7516 | -0.082 | -0.5264 | Yes |
| 236 | CYBRD1 |  |  | 7525 | -0.082 | -0.5251 | Yes |
| 237 | CRTAP |  |  | 7547 | -0.084 | -0.5253 | Yes |
| 238 | NID1 |  |  | 7561 | -0.084 | -0.5245 | Yes |
| 239 | KANK2 |  |  | 7565 | -0.084 | -0.5226 | Yes |
| 240 | SSR1 |  |  | 7569 | -0.085 | -0.5206 | Yes |
| 241 | PRDX6 |  |  | 7589 | -0.085 | -0.5205 | Yes |
| 242 | PHTF2 |  |  | 7595 | -0.086 | -0.5187 | Yes |
| 243 | SLC39A14 |  |  | 7617 | -0.087 | -0.5188 | Yes |
| 244 | NPTN |  |  | 7618 | -0.087 | -0.5164 | Yes |
| 245 | KDELR2 |  |  | 7622 | -0.087 | -0.5144 | Yes |
| 246 | BOC |  |  | 7635 | -0.087 | -0.5134 | Yes |
| 247 | P4HA1 |  |  | 7667 | -0.089 | -0.5147 | Yes |
| 248 | SLC35F5 |  |  | 7674 | -0.089 | -0.5129 | Yes |
| 249 | PLOD2 |  |  | 7718 | -0.092 | -0.5155 | Yes |
| 250 | INSIG1 |  |  | 7721 | -0.092 | -0.5132 | Yes |
| 251 | DKK3 |  |  | 7776 | -0.094 | -0.5170 | Yes |
| 252 | FBN1 |  |  | 7777 | -0.094 | -0.5145 | Yes |
| 253 | TNFRSF12A |  |  | 7784 | -0.094 | -0.5126 | Yes |
| 254 | CRISPLD1 |  |  | 7793 | -0.095 | -0.5109 | Yes |
| 255 | EDEM1 |  |  | 7798 | -0.096 | -0.5088 | Yes |
| 256 | ATP6V0E1 |  |  | 7802 | -0.096 | -0.5065 | Yes |
| 257 | ROBO1 |  |  | 7808 | -0.096 | -0.5044 | Yes |
| 258 | ELAVL1 |  |  | 7813 | -0.096 | -0.5023 | Yes |
| 259 | OSTC |  |  | 7826 | -0.097 | -0.5010 | Yes |
| 260 | ATP2B4 |  |  | 7858 | -0.099 | -0.5020 | Yes |
| 261 | EFEMP2 |  |  | 7869 | -0.099 | -0.5004 | Yes |
| 262 | CTSB |  |  | 7870 | -0.099 | -0.4977 | Yes |
| 263 | FUCA2 |  |  | 7872 | -0.100 | -0.4951 | Yes |
| 264 | CDH11 |  |  | 7885 | -0.101 | -0.4937 | Yes |
| 265 | TMEFF2 |  |  | 7965 | -0.105 | -0.5002 | Yes |
| 266 | DMD |  |  | 7992 | -0.107 | -0.5004 | Yes |
| 267 | GPC6 |  |  | 7999 | -0.107 | -0.4982 | Yes |
| 268 | SSR3 |  |  | 8023 | -0.109 | -0.4979 | Yes |
| 269 | ADAM19 |  |  | 8040 | -0.110 | -0.4968 | Yes |
| 270 | TRAM2 |  |  | 8079 | -0.112 | -0.4982 | Yes |
| 271 | FGFR1 |  |  | 8080 | -0.112 | -0.4951 | Yes |
| 272 | ATP1B1 |  |  | 8091 | -0.113 | -0.4932 | Yes |
| 273 | VCL |  |  | 8092 | -0.113 | -0.4901 | Yes |
| 274 | SPARCL1 |  |  | 8104 | -0.114 | -0.4883 | Yes |
| 275 | JAM3 |  |  | 8130 | -0.116 | -0.4881 | Yes |
| 276 | FLNA |  |  | 8171 | -0.119 | -0.4896 | Yes |
| 277 | HEXB |  |  | 8175 | -0.119 | -0.4866 | Yes |
| 278 | PON2 |  |  | 8177 | -0.120 | -0.4835 | Yes |
| 279 | CYR61 |  |  | 8178 | -0.120 | -0.4802 | Yes |
| 280 | RECK |  |  | 8210 | -0.122 | -0.4805 | Yes |
| 281 | IGF2R |  |  | 8211 | -0.122 | -0.4771 | Yes |
| 282 | CYFIP1 |  |  | 8224 | -0.124 | -0.4752 | Yes |
| 283 | LMNA |  |  | 8225 | -0.124 | -0.4717 | Yes |
| 284 | TWSG1 |  |  | 8229 | -0.124 | -0.4687 | Yes |
| 285 | HTRA1 |  |  | 8245 | -0.126 | -0.4670 | Yes |
| 286 | EGFR |  |  | 8251 | -0.126 | -0.4642 | Yes |
| 287 | COL6A2 |  |  | 8253 | -0.126 | -0.4608 | Yes |
| 288 | SEMA3C |  |  | 8293 | -0.131 | -0.4618 | Yes |
| 289 | CKAP4 |  |  | 8298 | -0.132 | -0.4587 | Yes |
| 290 | TRAM1 |  |  | 8301 | -0.132 | -0.4553 | Yes |
| 291 | SPARC |  |  | 8303 | -0.132 | -0.4518 | Yes |
| 292 | KDELR3 |  |  | 8317 | -0.134 | -0.4496 | Yes |
| 293 | TMEM50A |  |  | 8329 | -0.136 | -0.4472 | Yes |
| 294 | GSN |  |  | 8339 | -0.137 | -0.4445 | Yes |
| 295 | TM9SF2 |  |  | 8353 | -0.139 | -0.4422 | Yes |
| 296 | PTGFRN |  |  | 8360 | -0.141 | -0.4391 | Yes |
| 297 | FAM114A1 |  |  | 8364 | -0.141 | -0.4355 | Yes |
| 298 | CD164 |  |  | 8369 | -0.142 | -0.4321 | Yes |
| 299 | EXTL2 |  |  | 8371 | -0.143 | -0.4283 | Yes |
| 300 | FSTL1 |  |  | 8373 | -0.143 | -0.4245 | Yes |
| 301 | TM4SF1 |  |  | 8392 | -0.146 | -0.4226 | Yes |
| 302 | IL6ST |  |  | 8394 | -0.146 | -0.4187 | Yes |
| 303 | TPBG |  |  | 8401 | -0.146 | -0.4154 | Yes |
| 304 | EPHA3 |  |  | 8402 | -0.146 | -0.4114 | Yes |
| 305 | DNAJC3 |  |  | 8407 | -0.147 | -0.4078 | Yes |
| 306 | COL5A1 |  |  | 8428 | -0.150 | -0.4061 | Yes |
| 307 | ATP2B1 |  |  | 8436 | -0.150 | -0.4028 | Yes |
| 308 | ATXN1 |  |  | 8456 | -0.153 | -0.4008 | Yes |
| 309 | TXNDC12 |  |  | 8480 | -0.157 | -0.3992 | Yes |
| 310 | CD59 |  |  | 8508 | -0.163 | -0.3980 | Yes |
| 311 | COL5A2 |  |  | 8514 | -0.165 | -0.3940 | Yes |
| 312 | COL11A1 |  |  | 8518 | -0.165 | -0.3899 | Yes |
| 313 | SURF4 |  |  | 8519 | -0.166 | -0.3853 | Yes |
| 314 | COL4A2 |  |  | 8525 | -0.168 | -0.3813 | Yes |
| 315 | MAGT1 |  |  | 8551 | -0.173 | -0.3795 | Yes |
| 316 | ASPH |  |  | 8566 | -0.177 | -0.3763 | Yes |
| 317 | PLEKHH2 |  |  | 8589 | -0.186 | -0.3738 | Yes |
| 318 | MYDGF |  |  | 8591 | -0.186 | -0.3688 | Yes |
| 319 | RCN1 |  |  | 8602 | -0.191 | -0.3647 | Yes |
| 320 | LAMC1 |  |  | 8618 | -0.199 | -0.3611 | Yes |
| 321 | SPCS3 |  |  | 8633 | -0.204 | -0.3571 | Yes |
| 322 | LAMB1 |  |  | 8640 | -0.209 | -0.3521 | Yes |
| 323 | EMP1 |  |  | 8641 | -0.209 | -0.3464 | Yes |
| 324 | SERPINH1 |  |  | 8646 | -0.212 | -0.3410 | Yes |
| 325 | PDIA3 |  |  | 8647 | -0.212 | -0.3352 | Yes |
| 326 | CALD1 |  |  | 8649 | -0.213 | -0.3295 | Yes |
| 327 | ANXA1 |  |  | 8651 | -0.213 | -0.3237 | Yes |
| 328 | PRDX4 |  |  | 8660 | -0.217 | -0.3187 | Yes |
| 329 | COL4A1 |  |  | 8661 | -0.218 | -0.3127 | Yes |
| 330 | MANF |  |  | 8665 | -0.220 | -0.3071 | Yes |
| 331 | NES |  |  | 8677 | -0.227 | -0.3021 | Yes |
| 332 | FAT1 |  |  | 8679 | -0.228 | -0.2960 | Yes |
| 333 | SYNJ2 |  |  | 8680 | -0.228 | -0.2897 | Yes |
| 334 | LOXL2 |  |  | 8687 | -0.232 | -0.2841 | Yes |
| 335 | PLOD3 |  |  | 8693 | -0.236 | -0.2782 | Yes |
| 336 | COL6A1 |  |  | 8698 | -0.238 | -0.2721 | Yes |
| 337 | FKBP14 |  |  | 8700 | -0.238 | -0.2657 | Yes |
| 338 | ITM2B |  |  | 8705 | -0.242 | -0.2595 | Yes |
| 339 | DDOST |  |  | 8706 | -0.242 | -0.2528 | Yes |
| 340 | PXDN |  |  | 8708 | -0.244 | -0.2462 | Yes |
| 341 | PDIA4 |  |  | 8712 | -0.251 | -0.2397 | Yes |
| 342 | B2M |  |  | 8718 | -0.258 | -0.2332 | Yes |
| 343 | SYPL1 |  |  | 8724 | -0.261 | -0.2266 | Yes |
| 344 | NQO1 |  |  | 8725 | -0.262 | -0.2194 | Yes |
| 345 | PTPRG |  |  | 8726 | -0.263 | -0.2122 | Yes |
| 346 | LAPTM4A |  |  | 8730 | -0.267 | -0.2052 | Yes |
| 347 | ANXA2 |  |  | 8741 | -0.279 | -0.1987 | Yes |
| 348 | SRPX |  |  | 8746 | -0.285 | -0.1914 | Yes |
| 349 | TIMP1 |  |  | 8751 | -0.290 | -0.1839 | Yes |
| 350 | ADAM9 |  |  | 8756 | -0.296 | -0.1763 | Yes |
| 351 | APP |  |  | 8758 | -0.300 | -0.1681 | Yes |
| 352 | TMED9 |  |  | 8765 | -0.311 | -0.1603 | Yes |
| 353 | RRBP1 |  |  | 8768 | -0.320 | -0.1517 | Yes |
| 354 | GPX8 |  |  | 8771 | -0.324 | -0.1431 | Yes |
| 355 | CD63 |  |  | 8776 | -0.335 | -0.1343 | Yes |
| 356 | ITGB1 |  |  | 8777 | -0.335 | -0.1251 | Yes |
| 357 | CD44 |  |  | 8778 | -0.337 | -0.1159 | Yes |
| 358 | PPIB |  |  | 8782 | -0.351 | -0.1066 | Yes |
| 359 | CALU |  |  | 8784 | -0.362 | -0.0968 | Yes |
| 360 | HSPA5 |  |  | 8785 | -0.369 | -0.0866 | Yes |
| 361 | TNC |  |  | 8787 | -0.389 | -0.0761 | Yes |
| 362 | PTN |  |  | 8789 | -0.426 | -0.0645 | Yes |
| 363 | LMAN1 |  |  | 8790 | -0.441 | -0.0524 | Yes |
| 364 | CTSC |  |  | 8791 | -0.448 | -0.0401 | Yes |
| 365 | PDIA6 |  |  | 8792 | -0.448 | -0.0278 | Yes |
| 366 | HSP90B1 |  |  | 8794 | -0.487 | -0.0145 | Yes |
| 367 | POSTN |  |  | 8795 | -0.533 | 0.0001 | Yes |
Table: GSEA details [plain text format]

  

Fig 2: VANGRONINGEN\_MES: Random ES distribution      
 Gene set null distribution of ES for **VANGRONINGEN\_MES**
